# Integrative single‐cell sequencing analysis distinguishes survival‐associated cells from the breast cancer microenvironment

**DOI:** 10.1002/cam4.5892

**Published:** 2023-04-06

**Authors:** Ling Huang, Shijie Qin, Lingling Xia, Fei Ma, Liming Chen

**Affiliations:** ^1^ Department of Biochemistry, School of Life Sciences Nanjing Normal University Nanjing Jiangsu 210023 China; ^2^ Laboratory for Comparative Genomics and Bioinformatics, College of Life Science Nanjing Normal University Nanjing Jiangsu 210046 China; ^3^ Institute of Pediatrics, Shenzhen Children's Hospital Shenzhen Guangdong Province 518026 China; ^4^ Key Laboratory of Pathogen Microbiology and Immunology Institute of Microbiology, Chinese Academy of Sciences Beijing 100101 China

**Keywords:** breast cancer, immune evasion, prognostic cell, single‐cell sequencing, tumor microenvironment

## Abstract

**Background:**

Breast cancer shows a highly complex tumor microenvironment by containing various cell types. Identifying prognostic cell populations in the tumor microenvironment will improve the mechanistical understanding of breast cancer and facilitate the development of new breast cancer therapies by targeting the tumor microenvironment. The development of single‐cell sequencing reveals various cell types, states, and lineages within the context of heterogenous breast tumors, but identifying phenotype‐associated subpopulations is challenging.

**Results:**

Here, we applied Scissor (single‐cell identification of subpopulations with bulk Sample phenotype correlation) to integrate single cell and bulk data of breast cancer, and found that MHC‐deficient tumor cells, FABP5+ macrophages, and COL1A1+ cancer‐associated fibroblasts (CAFs) were detrimental to patient survival, while T cells and dendritic cells were the main protective cells. MHC‐deficient tumor cells show strong downregulation of MHC expression for immune evasion by downregulating interferon and JAK‐STATs signaling. FABP5+ macrophages show low antigen‐presenting activity via associating with lipid metabolism. Our data suggest that COL1A1+ CAFs may block T‐cell immune infiltration through cell interaction in breast tumor microenvironment.

**Conclusion:**

Taken together, our study reveals survival‐associated subpopulations in breast tumor microenvironment. Importantly, subpopulations related to immune evasion of breast cancer is uncovered.

## Graphical abstract

Integrating single cell data and bulk data to identify prognostic cells and their molecular characteristics related to survival of breast cancer.

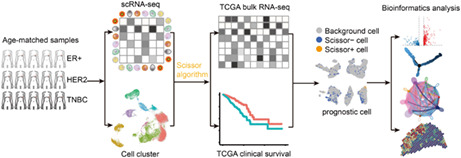

## 1 INTRODUCTION

Breast cancer is the most common malignant tumor and the main cause of cancer death in women.^1^ Clinically, breast cancer patients are widely divided into ER^+^ (Luminal A/Luminal B), HER2^+^ and triple negative breast cancer (TNBC) subtypes through estrogen receptor (ER), progesterone receptor (PR), and human epidermal growth factor receptor 2 (HER2).^2^, ^3^ Different breast cancer subtypes correspond to different treatment methods and prognosis.^4^, ^5^ Patients with HER2^+^ and TNBC subtypes had significantly lower survival and cure rates than those with ER^+^ subtypes.^4^, ^5^

Breast cancer occurs in the mammary epithelium with a complex tumor microenvironment.^5^, ^6^ Studies have reported significant heterogeneity of breast cancer cells, such as the malignancy and sensitivity to therapy of different tumor cell populations are significantly different.^7^, ^8^ Furthermore, tumor‐associated macrophages in the breast cancer microenvironment are thought to be associated with poor prognosis.^9^, ^10^ Conversely, high abundance of infiltrating lymphocytes in breast tumors is a marker of patient benefit from neoadjuvant chemotherapy.^11^ Stromal components such as cancer‐associated fibroblasts (CAFs) have also been widely reported to regulate breast cancer invasion and metastasis, extracellular matrix (ECM) remodeling, and lymphocyte infiltration.^10^, ^12^ Although there are emerging therapeutic potential of targeting breast tumor microenvironment, phenotype‐associated subpopulations in microenvironment represents a major challenge for exploiting tumor microenvironment to fight breast cancer.

Single‐cell sequencing technology has largely improved our understanding on cell types, states, and lineages within the context of heterogeneous tissues by capture genomic, transcriptomic, or proteomic information at the single‐cell level.^13^, ^14^ Applied to breast cancer, single‐cell sequencing has produced bulk and single‐cell sequencing.^5^, ^15^ However, current single‐cell data cannot directly link cell clusters with specific breast cancer phenotypes, such as disease grade, survival time, metastasis, and treatment outcome.^16^

In this study, we applied Scissor (single‐cell identification of subpopulations with bulk sample phenotype correlation) to analyze single‐cell data and large bulk transcriptome of breast cancer. Our results reveal phenotype‐associated subpopulations in breast tumor microenvironment, highlighting the critical roles of immune evasion‐related subpopulations and molecular mechanisms in breast cancer development.

## 2 MATERIALS AND METHODS

### 2.1 Data acquisition and preprocessing

TCGA (The Cancer Genome Atlas) primary breast cancer data were downloaded from UCSC Xena database (http://xena.ucsc.edu/). Only 1064 samples with clinical information and transcriptome were included. Breast cancer single‐cell transcriptome (GSE176078) was download in GEO database (https://www.ncbi.nlm.nih.gov/geo/).^15^ Only 15 samples with comparable age and high cell numbers were used, including five each of ER+, HER2+, and TNBC subtypes.

### 2.2 Single‐cell data processing and analysis

Single cell data were integrated using the Seurat R package (Version 4).^17^ Cells with less than 15% of mitochondrial genes, and genes in the range of 200–7500, and RNAs 500–100,000 were retained. The top 2000 highly variable genes were selected for subsequent cluster analysis. The appropriate principal components were selected by observing the cumulative error and significance, and different resolutions were set to determine the appropriate one for cell cluster. The Harmony R algorithm package was used to remove batch effects between samples to cluster the same cell type.^18^ Uniform Manifold Approximation and Projection (UMAP) was used to dimension reduction and visualize.^19^

### 2.3 Identification of malignant epithelial cells and normal epithelial cells

Potentially malignant epithelial cells were identified by inferring large‐scale copy number variations (CNVs) using InferCNV R package.^20^ B cells, T cells, and endothelial cells were used as normal reference cells. In order to reduce the computational burden and memory, only 500 reference cells were randomly selected, respectively, with repeatable random seed 123. Malignant and normal epithelial cells were distinguished by iterative clustering of CNVs.

### 2.4 Identification of prognostic cell types by Scissor

The Scissor R package was used to integrate breast cancer bulk and single‐cell data and overall survival information to identify cells associated with survival.^16^ Briefly, the Scissor algorithm first quantifies the similarity between single‐cell data and bulk data. Then Scissor optimized the regression model between the correlation matrix and the phenotype. Next, Scissor calculated the cells associated with the phenotype by imposing a sparsity penalty and graph regularization. Scissor's built‐in Cox regression model was used to calculate cells associated with overall survival. The prognostic cell ratio was determined by setting appropriate threshold of 0.03. The significance of prognostic cells was determined by performing a 100‐fold cross‐validation by Scissor's built‐in *reliability.test()*. For the survival phenotype, Scissor returned Scissor+ cells that were not conducive to patient survival, Scissor− cells that promoted patient survival, or background cells with no significant association.

### 2.5 Differential gene and gene set activity analysis

The Seurat's *Findmarkers()* were used to identify differential expression genes (DEGs), with |logFC| > 0.25 and FDR < 0.01. The gene sets activity of single cell was calculated by Seurat's *AddModuleScore()*. The hallmarks of cancer come from msigdb R package. The ClusterProfiler R package was used to enrich DEGs in Gene Ontology and Kyoto Encyclopedia of Genes and Genomes (KEGG).^21^ Multivariate Cox regression was used to calculate the risk contribution of genes to breast cancer survival after adjusting for age.

### 2.6 Cell communication analysis and spatial transcriptome analysis

The CellChat R package was used to calculate cell‐to‐cell communication.^22^ Only ligand and receptor pairs that were detected in at least 50 cells and *p*‐value <0.01 were retained. The spatial transcriptome data of breast cancer were downloaded from https://zenodo.org/record/4739739.YzaiA3ZBxD9,^15^ and were integrated using Seurat. Those spots with mitochondrial genes greater than 20% and genes less than 500 were filtered. After normalizing by the SCTransform, the spatial and single cell transcriptome were integrated to map cell types of single cells onto spatial locations.^17^

### 2.7 Statistical analysis

The Wilcoxon rank‐sum test was used to calculate the significance of gene or gene set enrichment scores and only when Bonferroni‐corrected *p*‐value <0.01 was considered significant. In cell communication analysis, only genes expressed in at least 50 cells and *p*‐value <0.01 were retained. The selection criterion of feature genes in pseudo‐time analysis was coefficient of variation FDR < 0.001. The significant threshold of gene enrichment analysis was *q*‐value <0.01. The significance threshold of survival‐related genes was *p*‐value <0.05. All analyses were performed on R software (version 4.0.0).

## 3 RESULTS

### 3.1 Breast cancer single‐cell atlas

**FIGURE 1:**
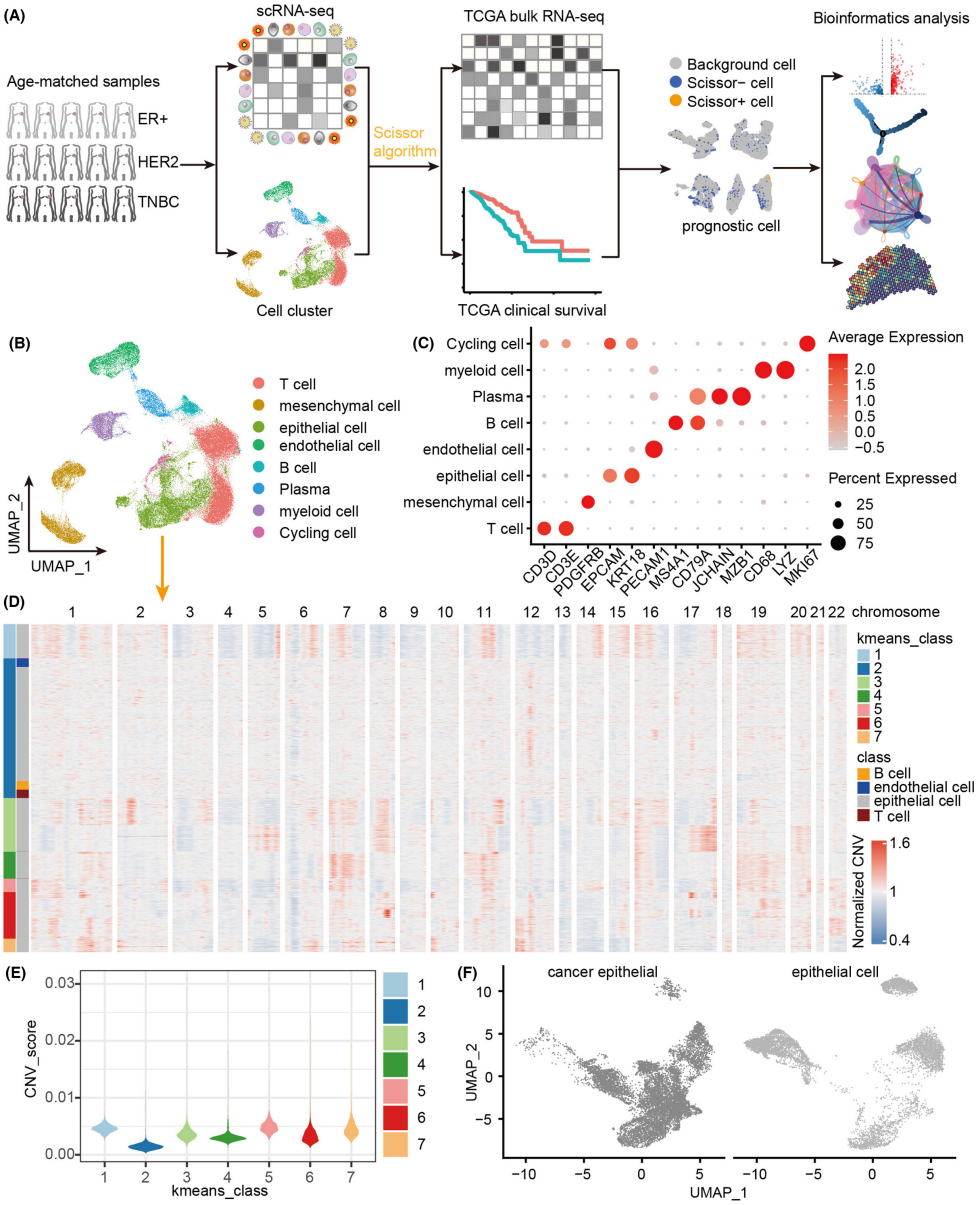
Breast cancer single‐cell atlas. (A) Flowchart of identifying prognostic cells using combined single‐cell and bulk data. (B) The UMAP cluster diagram shows the different cell types of breast cancer. (C) Bubble chart shows specific markers corresponding to different cell types. (D) Unsupervised cluster heat map of tumor epithelial and normal cells. (E) CNV score of all epithelial cells. (F) UMAP clustering of tumor epithelial cells normal epithelial cells.

To identify overall survival‐related cells in breast cancer patients, we employed the following analytical pipeline. Briefly, we selected single‐cell transcriptome from 15 cases containing three subtypes (Figure S1 and Table S1), and then combined them with bulk transcriptome by the Scissor algorithm (Figure 1A). We identified survival‐relevant Scissor+ or Scissor− cells in epithelial cells, immune cells, and stromal cells (Figure 1A). After quality control, we obtained 70,713 single cells in breast cancer (Figure 1B and Figure S1), including 26,473 T cells (CD3D+, CD3E+), 8872 mesenchymal cells (PDGFRB+), 17,515 epithelial cells (EPCAM+, KRT18+), 5992 endothelial cells (PECAM1+), 2928 B cells (MS4A1+, CD79A+), 3064 plasma cells (JCHAIN+, MZB1+), 4627 myeloid cells (CD68+, LYZ+), and 1224 proliferating cells (MKI67+) (Figure 1B,C). These cell types are adequately differentiated by the classical markers, illustrating the accuracy of cell type identification (Figure 1C and Figure S1). We further explored the heterogeneity of epithelial cells, and identified the neoplastic cells by copy number variation (CNV) (Figure 1D and Figure S2) and unsupervised clustering analysis (Figure 1E). Remarkably, the second cluster epithelial cells was considered normal epithelial cells because they were clustered together with normal reference cells (B cells, T cells, and endothelial cells) with the lowest CNV scores (Figure 1D,E), but the remaining epithelial cells could be classified as tumor cells accompanied by significantly higher CNV scores (Figure 1D,E). Ultimately, we found 10,916 tumor cells and 6618 normal epithelial cells (Figure 1F). Among them, normal epithelial cells and as a reference cell B cells, T cells, and endothelial cells, there is no obvious copy number abnormal area, but tumor cells have multiple areas of copy number increase or decrease area (Figure S2). Finally, the average proportion of tumor cells in 15 samples was 62.3%, which was consistent with the high tumor purity of breast tumor tissue.^16^

### 3.2 Identification of survival‐related cells in epithelial cells

**FIGURE 2:**
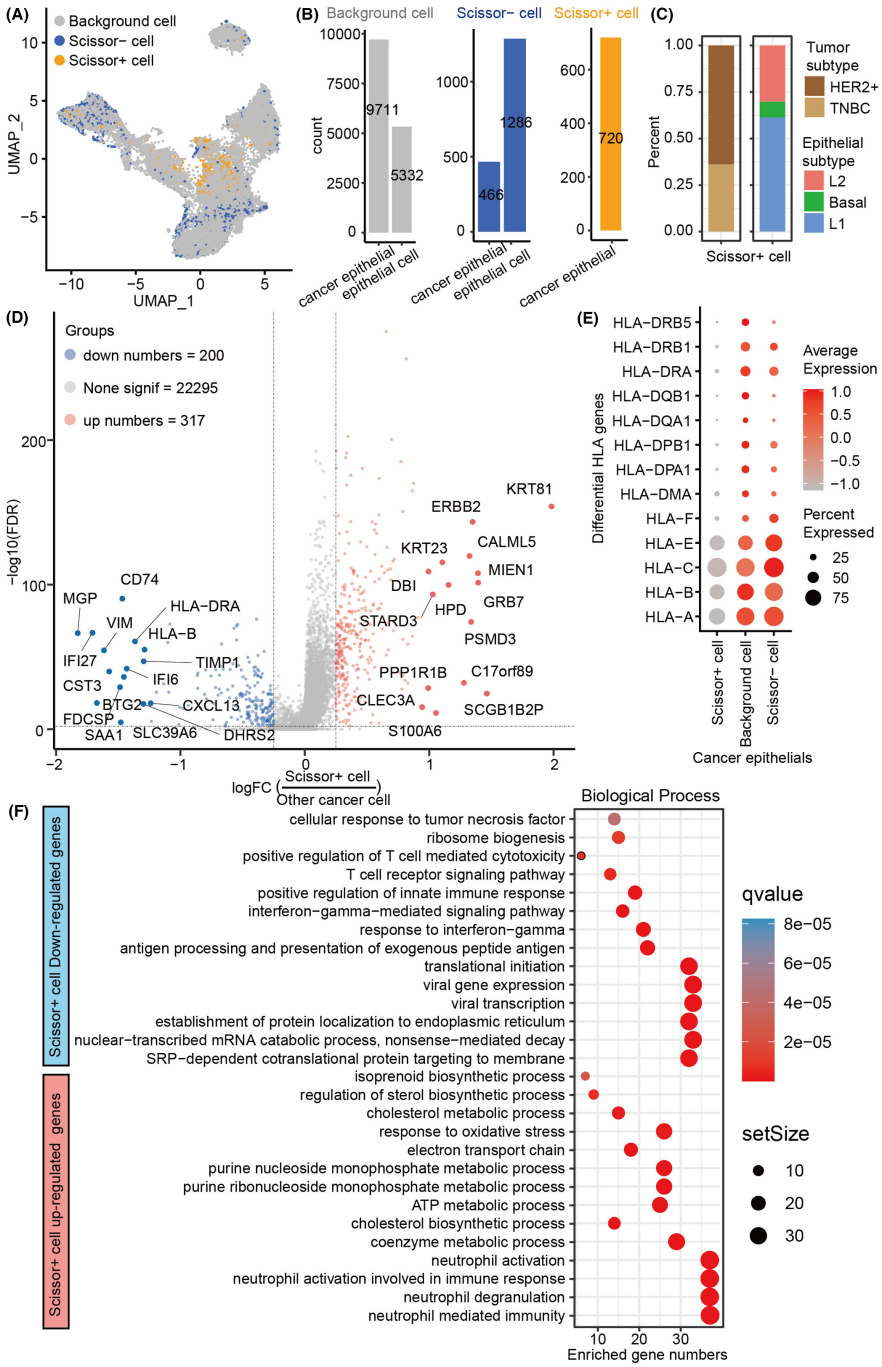
Identification of prognostic epithelial cells and their molecular characteristics. (A) Distribution of prognostic epithelial cell identified by the Scissor algorithm. (B) Number statistics and distribution of prognostic epithelial cell populations. (C) Proportion of Scissor+ prognostic cells in different subtypes and epithelial cell subsets. (D) DEGs between Scissor+ epithelial cells and other epithelial cells. (E) MHC expression level of Scissor+ epithelial cells. (F) Functional analysis of upregulated and downregulated DEGs in Scissor+ epithelial cells.

Here, we utilized the Scissor algorithm to further explore the heterogeneity of epithelial cells of breast cancer patients. As shown in Figure 2A, the Scissor algorithm divide cells into three categories: Scissor+ cells leading to worse patient survival, Scissor− cells promoting patient survival, and background cells not significantly associated.^16^ In total, we identified 720 Scissor+ and 1752 Scissor− epithelial cells as well as plenty of background epithelial cells (Figure 2A). Particularly, the 720 Scissor+ epithelial cells that led to the poor overall survival were all enriched in tumor cells, but the Scissor− epithelial cells that promote good survival were mainly enriched in normal epithelial cells (Figure 2B). Of note, previous studies showed that the ER+ subtype has the best prognosis, but the worse prognosis for HER2+ and TNBC subtypes.^2^, ^3^ Interestingly, we found that Scissor+ tumor cells were mainly concentrated in breast cancer patients with HER2+ and TNBC subtypes rather than the ER+ subtype (Figure 2C), revealing that the epithelial cell heterogeneity has a significant impact on patient survival. Besides, we further divided epithelial cells into three common subpopulations: basal cells (KRT14+, KRT5+), luminal 1 cells (SPLI+, PROM1+), and luminal 2 cells (ANKRD30A+, SYTL2+) (Figure S3),^23^ and found that Scissor+ tumor cells could be distributed in all three subpopulations, particularly the highest proportion of luminal 1 cells and the lowest proportion of basal cells (Figure 2C).

To reveal how Scissor+ tumor cells cause the poor overall survival in breast cancer patients, we further identified 200 upregulated and 317 downregulated DEGs in Scissor+ tumor cells and other tumor cells (Figure 2D and Table S2), finding that these top downregulated genes in Scissor+ tumor cells were *IFI6*, *BTG2*, *CD74*, *SLC39A6*, and *SAA1*, etc., while the top upregulated genes were *KRT81*, *SCGB1B2P*, *GRB7*, *MIEN1*, and *ERBB2* (also called HER2), etc. (Figure 2D). Remarkably, our results demonstrated that genes encoding major histocompatibility complex (MHC) in Scissor+ cells were all downregulated or even almost absent (Figure 2E), suggesting that Scissor+ cells may have extremely strong immune evasion. Further findings demonstrated that the downregulated DEGs in Scissor+ tumor cells are significantly enriched in biological processes such as “protein localization to the endoplasmic reticulum,” “antigen presentation and processing,” “T cell mediated cytotoxicity,” and “interferon production,” implying that Scissor+ cells have insufficient antigen presentation and thus evade immune surveillance. In contrast, these upregulated DEGs in Scissor+ tumor cells were significantly enriched in “neutrophil activation,” “oxidative stress,” and various metabolic processes, suggesting that Scissor+ tumor cells may only induce inflammatory immune response rather than T‐cell immunity, which may be caused by reprogramming the cellular metabolic process (Figure 2F).

### 3.3 JAK‐STATs signaling mediates immune escape of Scissor+ tumor cells

**FIGURE 3:**
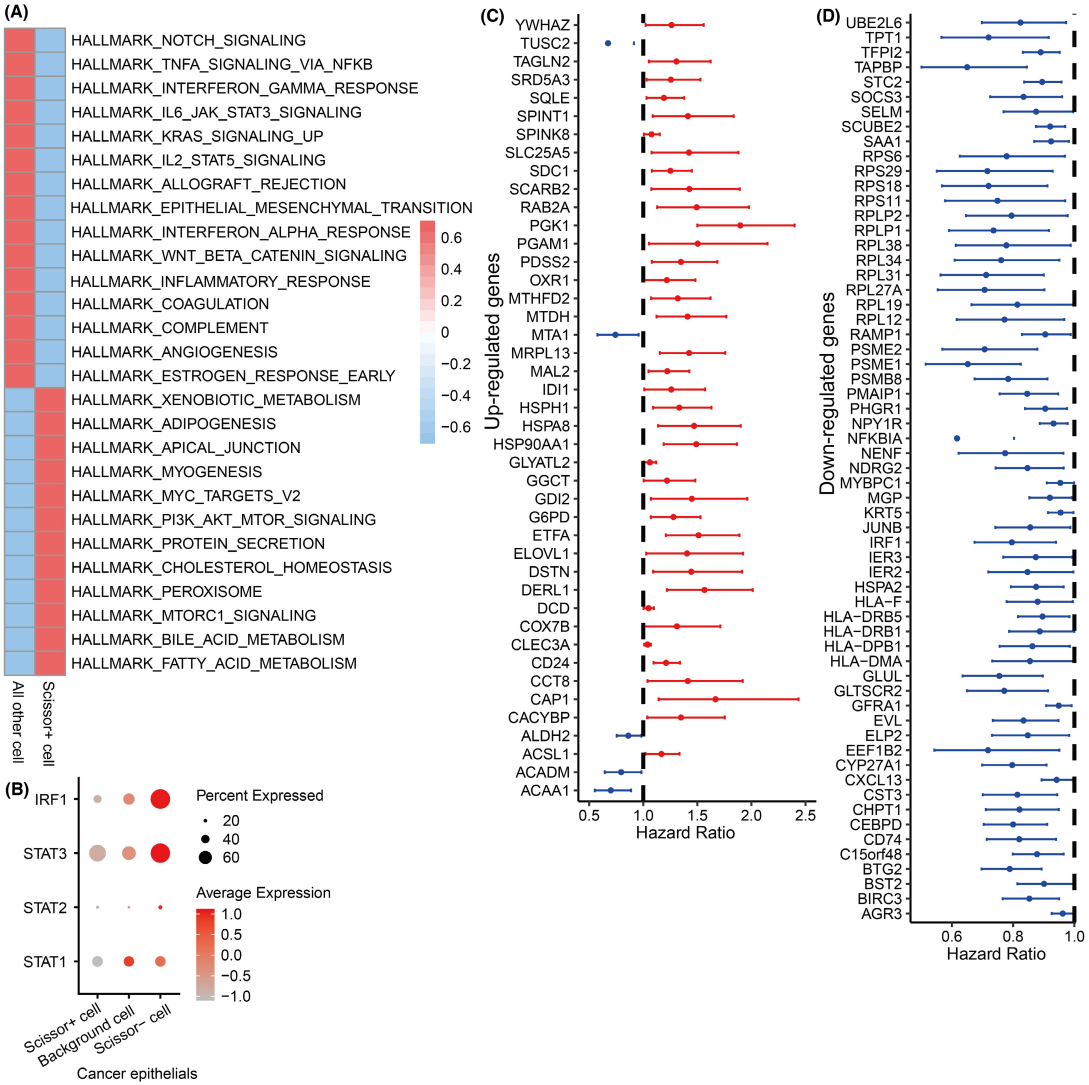
Interferon and JAK‐STATs signaling lead to immune escape in Scissor+ tumor cells. (A) The significant hallmarkers of cancer in Scissor+ tumor cells. (B) Differential expression of key transcription factors of JAK‐STATs pathway in Scissor+ tumor cells. (C) Significantly upregulated prognostic genes in Scissor+ tumor cells. (D) Significantly downregulated prognostic genes in Scissor+ tumor cells. A hazard ratio of more than 1 indicates that the gene is a risk factor for survival, while a hazard ratio of less than 1 indicates that the gene is a protective factor.

Herein, we further performed a transcriptome‐wide gene set activity analysis of 50 hallmarks of cancer, and found that these significantly activated hallmarks in Scissor+ tumor cells included “fatty acid metabolism,” “MTORC1 signaling,” “PI3K AKT signaling,” and “MYC target,” etc. (Figure 3A), which are usually promoting tumor progression. In contrast, these significantly downregulated hallmarks were “NOTCH signaling,” “TNFA signaling,” “Interferon alpha response,” “Interferon gamma response,” and “JAK‐STATs signaling” (Figure 3A), which are often related to the activation of antitumor and antiviral immunity. For example, interferon and JAK‐STATs signaling can induce the upregulated expression of the MHC genes (Figure 3B).^24^ Particularly, IRF1, STAT1, STAT2, and STAT3 transcription factors can directly activate the expression of MHCs.^24^ Interestingly, we found that IRF1, STAT1, STAT2, and STAT3 were all significantly downregulated in Scissor+ tumor cells compared to Scissor− and background tumor cells (Figure 3C). We also assessed the effects of DEGs in Scissor+ tumor cells on the survival of breast cancer patients. After adjusting for age, most upregulated DEGs were served as risk factors, such as YWHAZ, TAGLN2, SRD5A3, SQLE, SPINT1, and SDC1, which were unfavorable for the survival of patients (Figure 3D). Conversely, most downregulated DEGs were pro‐survival protective factors (Figure 3E). Interestingly, our results showed that many JAK‐STATs signaling as well as MHC components related molecules such as IRF1, HLA‐F, HLA‐DRB5, HLA‐DRB1, HLA‐DPB1, and HLA‐DMA can promote the survival of breast cancer patients^24^, ^25^, ^26^, ^27^, ^28^ (Figure 3E). Taken together, these results suggested that Scissor+ tumor cells (MHC‐deficient) not only may evade immune surveillance via downregulating interferon signaling and the JAK‐STATs pathway to reduce MHC (mainly includes MHC I and II) genes expression (Figure 3B), but also upregulate multiple risk factors and downregulate protective factors to cause the worse prognosis of breast cancer patients (Figure 3B).

### 3.4 Identification of prognostic cells in the immune microenvironment of breast tumors

**FIGURE 4:**
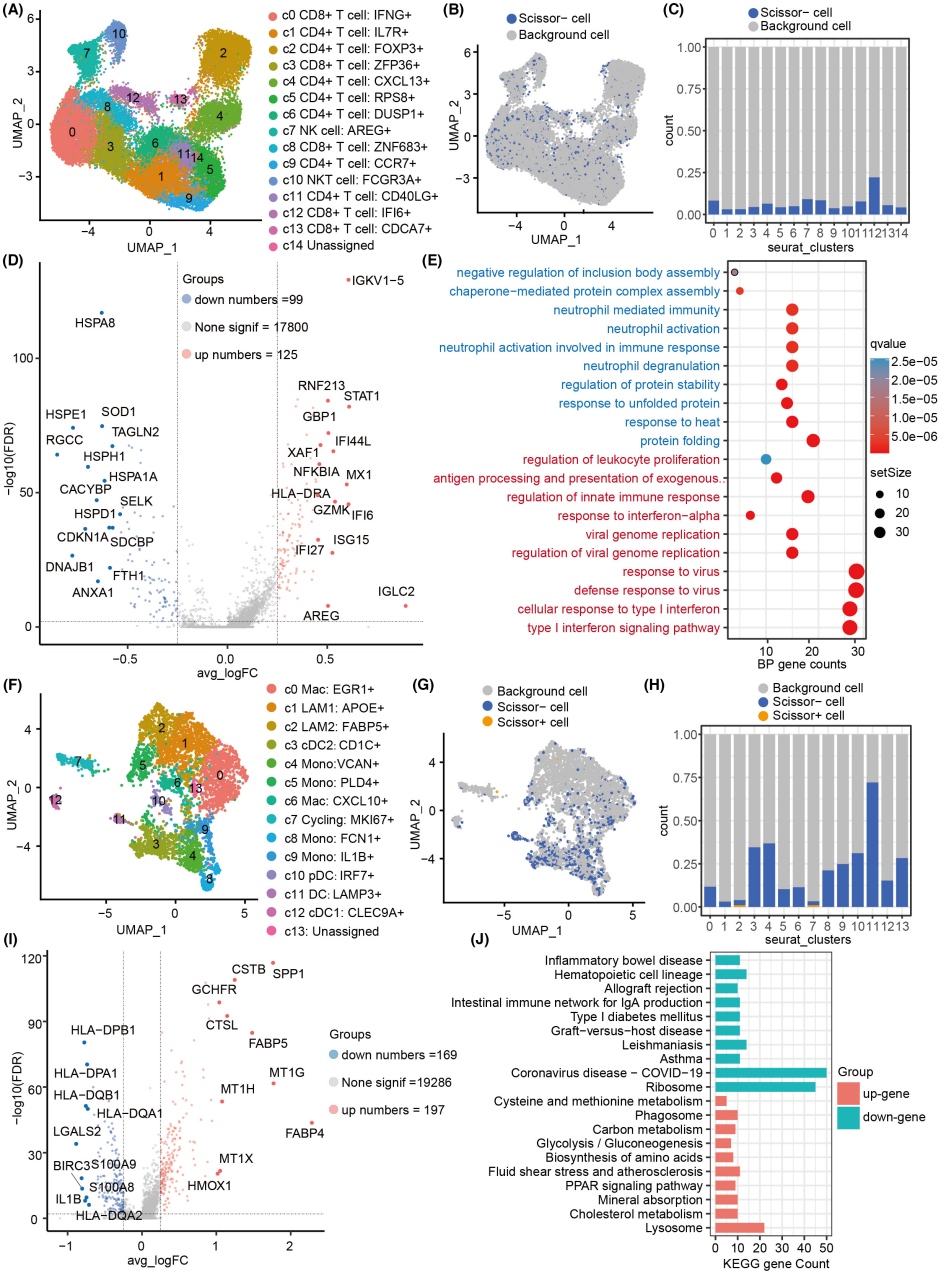
Scissor+/Scissor− prognostic cells in the immune microenvironment. (A) UMAP shows T‐cell subsets. (B) Identification of prognostic Scissor+/− T cells. (C) The proportion of Scissor+/− cells in different T cell subsets. (D) Volcano diagram showing the DEGs between scissor− T cells and the remaining T cells. (E) KEGG analysis of Scissor‐ T cell DEGs. (F) UMAP shows subsets of myeloid cells. (G) Identification of prognostic Scissor+/− myeloid cells. (H) The proportion of Scissor+/− cells in different myeloid cell subsets. (I) DEGs between FABP5+ macrophages and other macrophages. (J) KEGG analysis of FABP5+ macrophage DEGs.

Immune cells, especially myeloid cells representing innate immunity and T cells representing adaptive immunity, constitute the main components of the immune microenvironment of breast tumors.^29^ Here, we evaluated how T cells and myeloid cells, respectively, affect breast cancer survival using the Scissor algorithm. Results demonstrated that T cells could be subdivided into 14 clusters (Table S3), which mainly included CD4+ T cells, CD8+ T cells, NK cells, and NKT cells (Figure 4A). Of note, we identified Scissor− T cells that promoted the survival of breast cancer patients (Figure 4B), but no Scissor+ T cells were found, suggesting that T cells are the primary protective factors for patients. Further, our findings showed that these protective Scissor− T cells were spread across all different cell subsets (Figure 4C), such as IFI6+ CD8 T cells, AREG+ NK cells, ZNF683+ CD8 T cells, IFNG+ CD8 T cells, and CD40LG+ CD4 T cells (Figure 4A and Figure 4C), which have be proved to killing tumors.^30^, ^31^, ^32^, ^33^ Meanwhile, we found that Scissor− IL7R+ CD4 T cells and FOXP3+ CD4 T cells only accounted for a relatively small proportion (Figure 4A,C), which are generally considered to be less toxic to tumors because of their immature and inhibitory activity.^33^, ^34^ Additionally, DEGs analysis showed 125 upregulated genes such as IGLC2, STAT1, IFI6, IGKV1‐5, MX1, GZMK, and IFI44L in Scissor− T cells, as well as 99 downregulated genes such as RGCC, DNAJB1, HSPE1, CDKN1A, HSPH1, CACYBP, and ANXA1, etc. (Figure 4D and Table S4). These upregulated genes were mainly enriched in the pathways related to “regulation of leukocyte proliferation,” “regulation of innate immune response,” “type I interferon,” “antiviral,” and “antigen presentation and leukocyte proliferation” (Figure 4E), but these downregulated genes were mainly enriched in “protein folding and stability” and “neutrophil activity” (Figure 4E). Together, our results suggest that T cells should be major protective factor in breast cancer patients, particularly these activated T cells.

Myeloid cells were also re‐clustered into 13 subgroups (Table S5), which mainly included M1‐like macrophages (Mac: CXCL10+) and M2‐like macrophages (Mac: EGR1+), monocytes (Mono), dendritic cells (DC), plasmacytoid dendritic cells (pDC), and so on (Figure 4F). Besides, we identified two clusters of myeloid cells with highly expressed genes that were involved in fatty acid synthesis (APOE+ and FABP5+) (Figure 4F), and found that multiple myeloid cells with a large number of Scissor− cells may be acted as protective factors for the survival of breast cancer patients (Figure 4G), especially dendritic cells had the highest percentage, which is consistent with their roles in promoting tumor killing by presenting antigen. Of note, some Scissor− monocytes (e.g., Mono: VCAN+ and Mono: IL1B+) had also protective effects on the survival of patients. Moreover, although we only found seven Scissor+ myeloid cells, they were predominantly FABP5+ macrophages (5/7) (Figure 4G,H). Interestingly, previous reports have indicated that FABP5+ macrophages are likely to be a lipid‐associated macrophages (LAM) with similar characteristics to obese mice and human,^35^ particularly, the FABP5 overexpression is detrimental to the survival of breast cancer patients.^15^ To further explore the underlying reasons why FABP5+ macrophages are not conducive to survival, we calculated the DEGs. Compared with other myeloid cells, we found 197 upregulated genes such as FABP4, MT1G, SPP1, FABP5, CSTB, CTSL, and MT1H, but 169 downregulated genes including LGALS2, S100A9, BIRC3, HLA‐DPB1, IL1B, HLA‐DQB1, and HLA‐DPA in FABP5+ macrophages (Figure 4I and Table S6). Functional analysis demonstrated that these upregulated genes were significantly enriched in processes related to “lysosomes,” “Glycolysis/Gluconeogenesis” and a variety of metabolic, while these downregulated genes were enriched in “Ribosome,” “Graft‐versus‐host disease,” “Type I Diabetes Mellitus,” “Intestinal immune network for IgA production,” and “Allograft rejection” (Figure 4J). Together, our results suggested that FABP5+ macrophages may be an emerging dangerous target cells in the breast tumor microenvironment in addition to the majority of macrophages that exert normal protective effects.

### 3.5 Identification of prognostic cells in the stromal microenvironment of breast tumors

**FIGURE 5:**
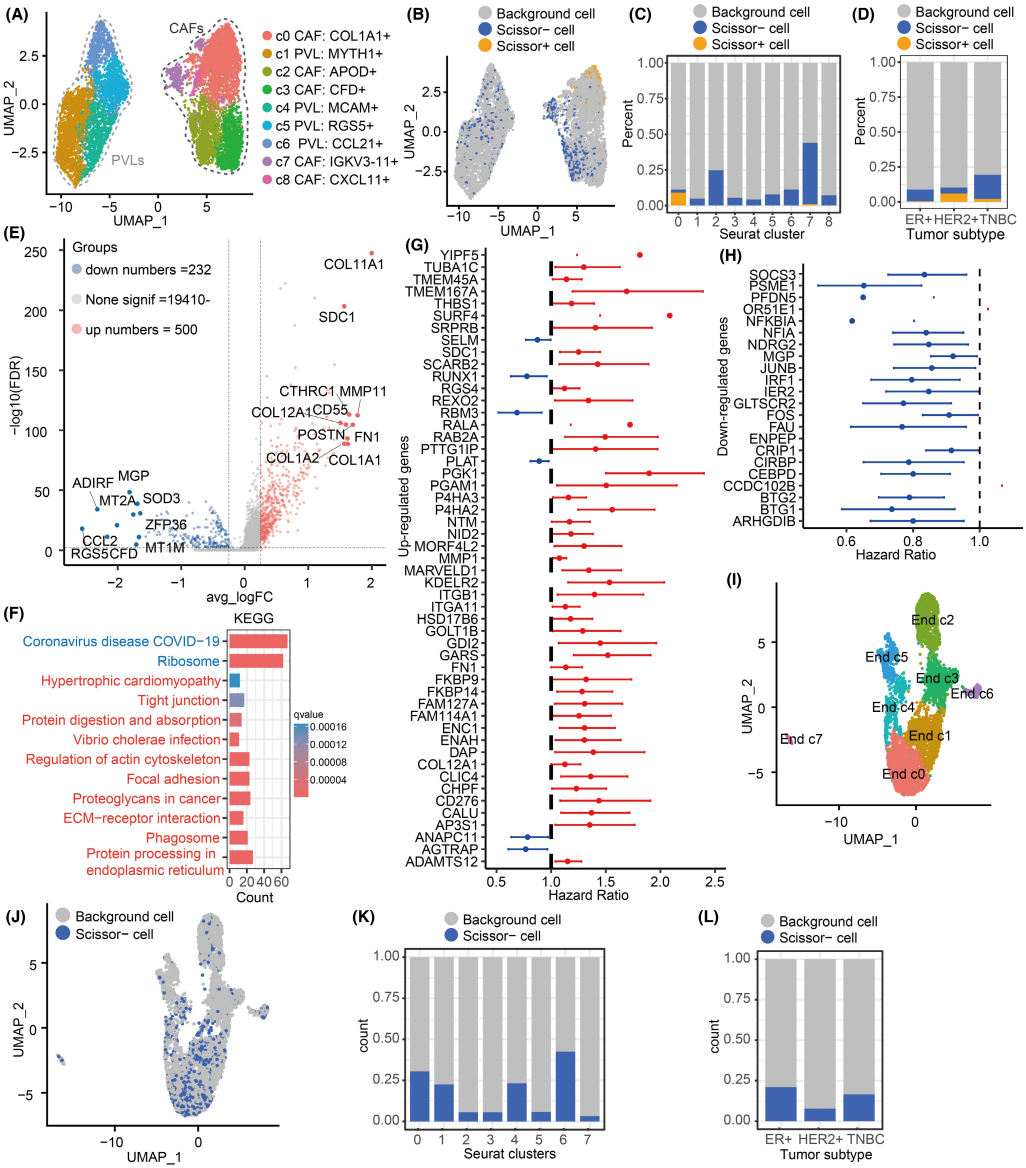
Scissor+/Scissor− prognostic cells in the stromal microenvironment. (A) UMAP shows mesenchymal cell subsets. (B) Identification of prognostic Scissor+/− mesenchymal cells. (C) The proportion of Scissor+/− cells in different mesenchymal cell subsets. (D) Proportion of Scissor+/− cells in ER+, HER2+, and TNBC subtypes. (E) Volcano diagram showing the DEGs between Scissor+ mesenchymal cells and the remaining cells. (F) KEGG enrichment analysis of Scissor+ mesenchymal DEGs. (G) Significantly upregulated prognostic genes in Scissor+ mesenchymal cells. (H) Significantly downregulated prognostic genes in Scissor+ mesenchymal cells. (I) UMAP displays endothelial cell subsets. (J) Identification of prognostic Scissor+/− endothelial cells. (K) Proportion of scissor− endothelial cells in different endothelial cell subsets. (L) Proportion of scissor− endothelial cells in ER+, HER2+, and TNBC subtypes.

Stromal microenvironment is commonly thought to help tumor cells obtain nutrients, spread and shield immune cell infiltration; particularly, stromal cells are also highly heterogeneous. Thus, we herein re‐clustered stromal mesenchymal cells into nine clusters, including five clusters of CAFs and four clusters of perivascular like cells (PVLs) (Figure 5A and Table S7), and identified 192 Scissor+ cells and 793 Scissor− mesenchymal cells (Figure 5B). Interestingly, these Scissor+ cells were found to mainly concentrate in COL1A1+ CAFs (Figure 5A,C), more importantly, Scissor+ CAFs were also found to mainly concentrate in patients with HER2+ and TNBC subtypes, implying that these COL1A1+ CAFs could contribute to their refractory and the poor prognosis for patients (Figure 5D). In addition, we also found that a small number of IGKV3‐11+ CAFs also showed adverse effects on the survival of breast cancer patients. Although the proportion is relatively small, whether they affect the survival rate of patients is worthy of further verification, because they may be new prognostic CAFs with no report. Similarly, we identified 500 upregulated genes such as COL11A1, MMP11, POSTN, CTHRC1, COL1A1, FN1, COL12A1, and SDC1, as well as 232 downregulated genes such as RGS5, ADIRF, CCL2, MGP, MT2A, and CFD in COL1A1+ CAFs (Figure 5E and Table S8). Functional analysis showed that these upregulated DEGs in COL1A1+ CAFs were significantly enriched in “Protein processing in endoplasmic reticulum,” “Tight junction,” “ECM‐receptor interaction,” “Proteoglycans in cancer,” “Focal adhesion,” and “Regulation of actin cytoskeleton,” etc. (Figure 5F), but these downregulated DEGs were only enriched in “ribosomes” (Figure 5F). Herein, we further detected how these DEGs in Scissor+ CAFs affected the survival of breast cancer patients, finding that most upregulated DEGs were risk factors (Figure 5G), while almost all downregulated DEGs were protective factors, especially ribosomal family genes such as RPS9, RPS8, RPS6, RPL38, and RPL34 (Figure 5H). Collectively, our results suggested that COL1A1+ CAFs may be the main dangerous cells in the stromal microenvironment, which could promote tumorigenesis and lead to poor prognosis for breast cancer patients.

In this work, we also re‐clustered these endothelial cells into eight subgroups (Figure 5I and Table S9). However, we did not find Scissor+ cells in endothelial cells causing the poor prognosis of breast cancer patients (Figure 5J), suggesting that endothelial cells may not be involved in tumorigenesis. In contrast, we identified multiple Scissor− cells across different endothelial cell subsets, implying that Scissor− cells may be contributed to the good survival of breast cancer patients (Figure 5K). Especially, we found that these Scissor− cells were most prevalent in ER+ subtypes with better prognosis, but least in HER2+ subtypes (Figure 5L). Together, our results suggested that endothelial cells in the stroma are unlikely to contribute to the poor survival of breast cancer patients.

### 3.6 Prognostic cell communication analysis reveals cell–cell interaction targets

**FIGURE 6:**
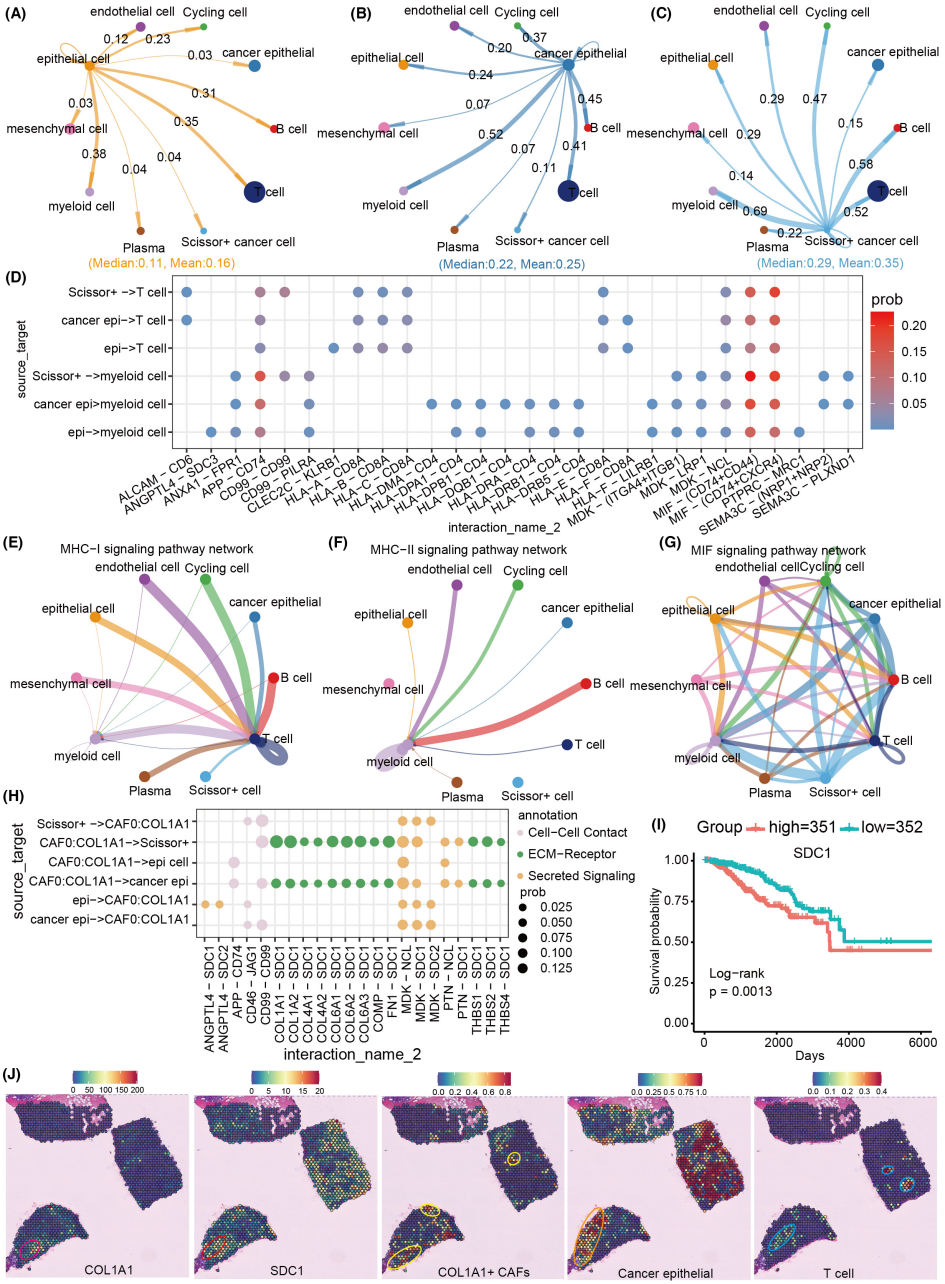
Cell‐to‐cell communication between breast cancer prognostic cells. (A–C) Cell communication frequency of normal epithelial cells, tumor cells, and Scissor+ tumor cells. (D) Significantly altered ligand and receptor pairs between epithelial and immune cells. (E–G) Distribution of cell communication networks mediated by MHC‐I, MHC‐II, and MIF signaling pathways in different cell types. The line color represents different cell–cell interactions, and the line thickness represents the frequency of cell–cell interactions. The thicker the line, the stronger the cell–cell interaction, and the thinner the line, the weaker the cell–cell interaction. (H) Ligand and receptor pairs that mediate specific interactions between COL1A1+ CAFs and epithelial cells. (I) Survival rate of breast cancer patients in high and low SDC1 expression group. (J) Spatial distribution of COL1A1, SDC1 gene and COL1A1+ CAFs, cancer cells and T cells in TNBC breast cancer tissues. Each spot in the figure records specific spatial location information, and each spot may contain from one to a dozen cells. The color of each spot represents the amount of gene expression in it or the predictive percentage score for each cell type. The locations marked by circles are areas of spatial co‐localization.

Cell‐to‐cell communication in tumor microenvironment can influence tumor occurrence and spread. Thus, we further explored how frequently normal epithelial cells, tumor cells, and Scissor+ tumor cells communicate with other cells (Figure S4A). Compared with normal epithelial cells, we found that the total frequency of cell communication between tumor cells and other cells was significantly increased (Median: 0.11 vs. Median: 0.22, Mean: 0.16 vs. Mean: 0.25), and Scissor+ tumor cells increased more (Median: 0.29, Mean: 0.35) (Figure 6A–C). Interestingly, normal cells, tumor cells, and Scissor+ tumor cells showed an increasing trend of gradient in interactions with myeloid cells, T cells, and mesenchymal cells (Figure 6A–C), implying that cancerous cells may need more interactions to domesticate surrounding cells for promoting their own exist, particularly Scissor+ tumor cells may have a stronger influence on cell communication (Figure S4A). Remarkably, our results demonstrated detailed ligands and receptors (Figure S4B), and found that CD99‐CD99 could mediate the interaction between Scissor+ tumor cells, myeloid cells, and T cells (Figure 6D), as well as MHC I‐mediated antigen presentation interaction was significantly decreased in tumor cells and Scissor+ tumor cells, especially MHC II‐mediated antigen presentation was absent in Scissor+ tumor cells (Figure 6D). In contrast, we found that inflammatory interactions between tumor cells or Scissor+ tumor cells and myeloid cells were significant increased, such as APP‐CD74, and MIF‐(CD74 + CD44) (Figure 6D). These above results could be also validated by the interaction network of a signaling pathway level, that is, MHC‐I and MHC‐II signaling led to the interaction of Scissor+ cells with T cells and myeloid cells at a decreased frequency, while the inflammatory MIF signaling resulted in an increased frequency (Figure 6E–G). Furthermore, we used spatial transcriptome data to prove whether these ligand–receptor pairs are spatially localized. Our results confirmed the spatial proximity of multiple sets of ligands and receptors, such as ANXA1‐FPR1, APP‐CD74, MIF‐(CD74 + CD44), and CD99‐CD99, and they basically correspond to the location of tumor cells, myeloid cells, and T cells, respectively (Figure S5). Interestingly, we also identified several interactions between COL1A1+ CAFs and all epithelial cells, which could be specific for tumor cells and Scissor+ tumor cells, and be highly dependent on the SDC1 receptor (Figure 6H). We found that the interactions between COL1A1+ CAFs and tumor cells and Scissor+ tumor cells were mainly mediated by collagen, including COL1A1‐SDC1, COL1A2‐SDC1, COL4A1‐SDC1, COL4A2‐SDC1, COL6A2‐SDC1, and COL6A2‐SDC1, especially Scissor+ tumor cells had stronger interaction with COL1A1 + CAFs (Figure 6H). Importantly, our results showed that breast cancer patients with highly expressed SDC1 had lower overall survival than those with low SDC1 expression (Figure 6I), suggesting that SDC1 may be a target for harmful cellular interactions. Meanwhile, the spatial transcriptome also demonstrated that COL1A1 and SDC1 co‐localized in spatially adjacent COL1A1+ CAFs and breast cancer cells (Figure 6J), particularly the interaction between COL1A1+ CAFs and breast cancer cells was negatively correlated with the distribution of T cells to some extent (Figure 6J), suggesting that COL1A1+ CAFs could hinder the infiltration of T cells into cancer cells. Together, our results suggested that COL1A1+ CAFs and Scissor+ tumor cells are harmful and can inhibit immune infiltration through SDC1 receptor interaction and lead to poor prognosis of breast cancer patients.

## 4 DISCUSSION

It is necessary to reveal the cell population in the breast cancer microenvironment that contributes to the survival of patients for prolonging their life span and treatment. Scissor is a recently developed algorithm, which identifies biologically and clinically relevant cell subpopulations from single‐cell assays by leveraging phenotype and bulk‐omics datasets.^16^ Applied scissor to breast cancer, we identified 720 Scissor+ tumor cell populations in epithelial cells that contribute to poor survival in breast cancer patients. These highly harmful Scissor+ tumor cells can downregulate interferon and JAK‐STATs signaling to reduce MHC‐mediated antigen presentation to evade immune surveillance (Figure 2 and Figure 3).^24^ Second, we found that novel FABP5+ macrophages are detrimental to breast cancer survival rather than M2‐like macrophages that have been repeatedly reported.^35^ FABP5+ macrophages are likely to be a LAM, which are generally not conducive to the prognosis of patients.^36^ Interestingly, previous studies found that LAM in the mouse model has immunosuppressive activity and can promote tumor occurrence or lung metastasis.^37^, ^38^ In addition, Liu et al. also demonstrated that LAM in breast cancer samples is increased, and depletion of LAM is conducive to survival and anti PD1 treatment.^39^ Importantly, breast tissue itself is rich in lipids, so it is reasonable to believe that FABP5+ LAM may be a new tumor target following M2 like macrophages, especially in breast cancer. Third, our study identified COL1A1+ CAFs as the major cell population detrimental to patient survival in the stromal environment (Figure 5). COL1A1+ CAFs extremely expressed a large amount of collagen and fibronectin, and were highly enriched in adhesions, extracellular matrix, and tight junction pathway that are associated with extracellular matrix remodeling, tumor cell metastasis, and obstruction of immune cell infiltration (Figure 5).^10^, ^12^

We also found that these prognostic cell populations described above have close cell‐to‐cell interactions with surrounding cells. Specifically, our results showed that the cancerous cells had more cell‐to‐cell interactions than the normal cells, especially the Scissor+ tumor cells with the highest degree of malignancy (Figure 6). These interactions may be the basis for cancer cells to domesticate the cells of the microenvironment to promote self‐adaptation. We noted that Scissor+ tumor cells have interaction with macrophages and T cells through CD99 that was upregulated in breast tumor cells (Figure 6 and Table S2). Interestingly, CD99 is an adhesion molecule, which is mainly upregulated in human Ewing sarcoma,^40^ and was a potential therapeutic target for hematological malignancies.^41^ In addition, anti‐CD99 CAR‐T cells can specifically recognize CD99 antigen on tumor cells, and effectively inhibit the proliferation and induce apoptosis of tumor cells.^42^ We hypothesize that CD99‐mediated cell interactions may inhibit immune cells through some unknown mechanism, and therefore, it may be an effective target of breast cancer. In addition, we noted that Scissor+ tumor cells downregulated MHC I and MHC II signaling and upregulated inflammatory MIF signaling‐mediated cellular interactions (Figure 6). Combined with functional analyses, we concluded that more dangerous Scissor+ tumor cells may induce inflammatory myeloid rather than T‐cell immune activity through reprogramming metabolic pathways (Figures 3 and 6). Finally, we also found that COL1A1+ CAFs mediated the interaction with tumor cells through collagen proteins (Figure 6). Importantly, these interactions are likely to surround tumor cells with a large number of stromal cells, which can largely isolate T‐cell infiltration into the tumor site.^43^, ^44^ Therefore, targeting the interaction between COL1A1+ CAFs and tumor cells may be an effective treatment for breast cancer. In particular, we found that SDC1 was a key receptor mediating COL1A1+ CAFs and cancer cells, and it was significantly upregulated in both cell types (Figures 5 and 6 and Table S2). Previous studies have reported that SDC1 is a marker of epithelial–mesenchymal transition,^45^ and its overexpression can promote pancreatic and breast cancer.^46^ Chen et al. reported that the CCL5‐SDC1/4 interaction between T cells and tumor cells can promote pancreatic cancer metastasis^47^ and the monoclonal antibody SDC1 against pancreatic cancer is being actively developed.^48^ In conclusion, we believe that SDC1 may be a potential target for blocking the interaction between COL1A1+ CAFs and tumor cells to promote immune infiltration in breast cancer.

We also compared the distribution of these dangerous and protective prognostic cells among breast cancer subtypes. Interestingly, we found that the distribution of Scissor+ tumor cells, FABP5+ macrophages, and COL1A1+ CAFs in different subtypes corresponded with their clinical prognostic outcomes (Figures 2, 4 and 5). Overall, hazardous Scissor+ cells were concentrated in HER2+ and TNBC subtypes, while protective Scissor− cells were enriched in ER+ subtypes. Considering that the current subtypes of breast cancer, especially TNBC and HER2+, still have some limitations in prognosis prediction and treatment, we would like to propose a new concept that combined prognostic cells with existing subtype classification of patients may assist more effective prediction methods and treatment guideline of breast cancer.

In summary, integrating single‐cell data with phenotype information to dissect clinically significant subsets from heterogeneous cell populations in breast tumors, we unravel the most survival‐relevant cell subpopulations in tumor microenvironment for future targeting breast cancer by exploiting tumor microenvironment. Importantly, our results raise potential cell subpopulations and molecules as targets to further development of effective immunotherapies to fight breast cancer with limited treatment options, such as triple‐negative breast cancer (TNBC).

## AUTHOR CONTRIBUTIONS

**Ling Huang:** Data curation (lead); formal analysis (lead); methodology (lead); visualization (equal); writing – original draft (lead); writing – review and editing (equal). **Shijie Qin:** Methodology (equal); visualization (equal); writing – original draft (equal); writing – review and editing (equal). **Lingling Xia:** Data curation (equal); methodology (equal). **Fei Ma:** Writing – original draft (equal); writing – review and editing (equal). **Liming Chen:** Funding acquisition (supporting); project administration (supporting); writing – review and editing (equal).

## FUNDING INFORMATION

This study was funded by National Natural Science Foundation of China (Grant No.: 81974447 to L.C.), Natural Science Foundation of Jiangsu Province (Grant No.: SBK2020010058 to L. C.), Project funded by China Postdoctoral Science Foundation (Grant No.: 2022M723344 to S. Q.), and the Priority Academic Program Development of Jiangsu Higher Education Institutions.

## CONFLICT OF INTEREST STATEMENT

No competing interest is declared.

## ETHICS STATEMENT

All participants provided written informed consent where appropriate and this study was approved by the relevant research institutional review.

## Supporting information


Figure S1‐S5

Table S1‐S5

## Data Availability

All data can be found in Materials and Methods. The analysis code can be obtained from author under the rationality.
