# Supplementary material for: Integrative single‐cell sequencing analysis distinguishes survival‐associated cells from the breast cancer microenvironment
Source: Cancer Med. 2023 Apr 6;12(11):12896–911. doi: 10.1002/cam4.5892 (PMC10278529; doi:10.1002/cam4.5892)
Supplement: Supplementary file 1 — Figure S1‐S5 [file CAM4-12-12896-s002.pdf]

Supplementary information for

Combining single-cell and bulk transcriptomes reveals prognostic cells in the breast cancer microenvironment

Authors: Ling Huang[1#], Shijie Qin[2,3,4#], Lingling Xia[1], Fei Ma[2]*, Liming Chen[1]*

Correspondence to: Liming Chen, chenliming1981@njnu.edu.cn (L. C.) or mafei01@tsinghua.org.cn (F.M.).

This file includes:

Figure S1 to S5

Other Supplementary Materials for this manuscript include the following:

Table S1 to S9

Table S1: Basic clinical and single cell information of 15 breast cancer samples included in the analysis.

Table S2: Differentially expressed genes between Scissor+ cancer epithelial cells and other cancer epithelial cells.

Table S3: Top 50 up-regulated genes corresponding to different T cell subsets.

Table S4: Differentially expressed genes between Scissor- T cell population and remaining T cells.

Table S5: Top 50 up-regulated genes corresponding to different myeloid cell subsets.

Table S6: Differentially expressed genes between Scissor+ FABP5+ macrophage population and remaining myeloid cells.

Table S7: Top 50 up-regulated genes corresponding to different mesenchymal cells subsets.

Table S8: Differentially expressed genes between Scissor+ CAFs population and remaining mesenchymal cells.

Table S9: Top 50 up-regulated genes corresponding to different endothelial cells subsets.

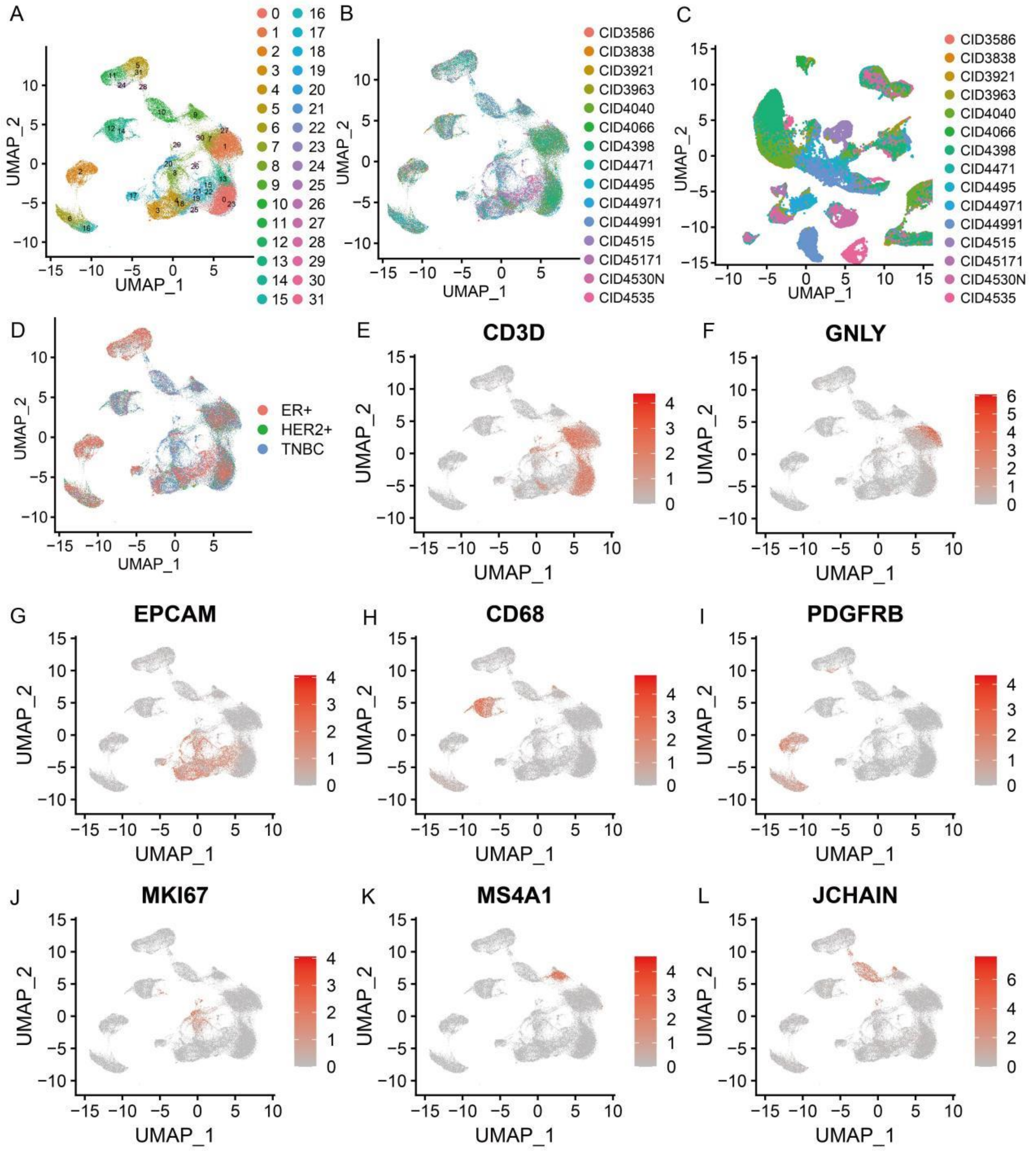

Figure S1. Breast cancer single cell map with sample and marker information.

A: UMAP shows that breast cancer tissue is clustered into 31 clusters of cells after removing the batch. B: UMAP distribution of single cells in different samples after removing the batch. C: UMAP distribution of single cells in different samples before removing the batch. The Harmony R algorithm package was used to remove batch effects between samples to cluster the same cell type. D: Distribution of single cells of

different subtypes in UMAP after removing the batch. E~L: Expression of specific markers corresponding to different cell types of breast cancer.

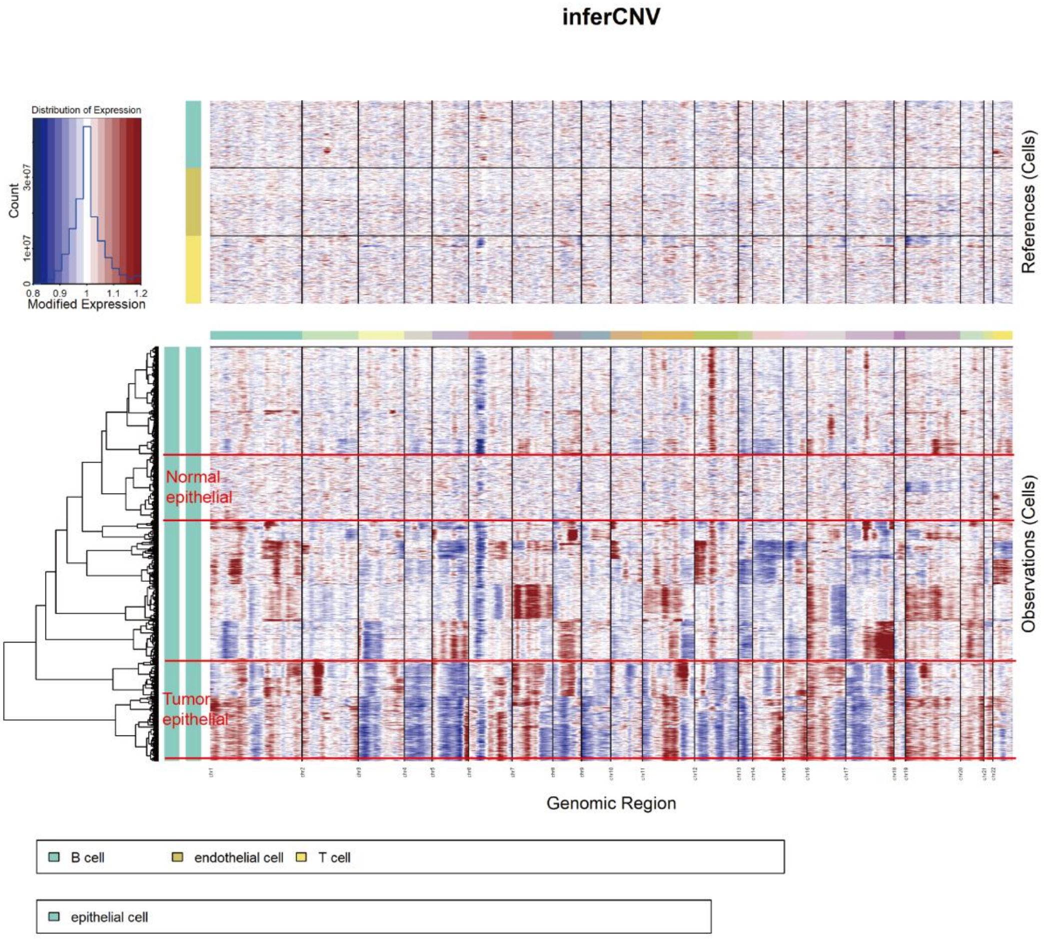

Figure S2. Large-scale copy number variation calculations to distinguish breast cancer epithelial cells from normal epithelial cells.

B cells, T cells and endothelial cells were taken as reference cells with normal copy number, and the copy number scores of all epithelial cells were recursively clustered to distinguish cancerous epithelial cells from normal epithelial cells. As can be seen in the upper part of the figure, as a reference cell B cells, T cells and endothelial cells did not

appear significant copy number increase or copy number reduction area. In the lower half of the graph, cells that show a large increase or loss of copy number compared to normal epithelium are considered tumor cell.

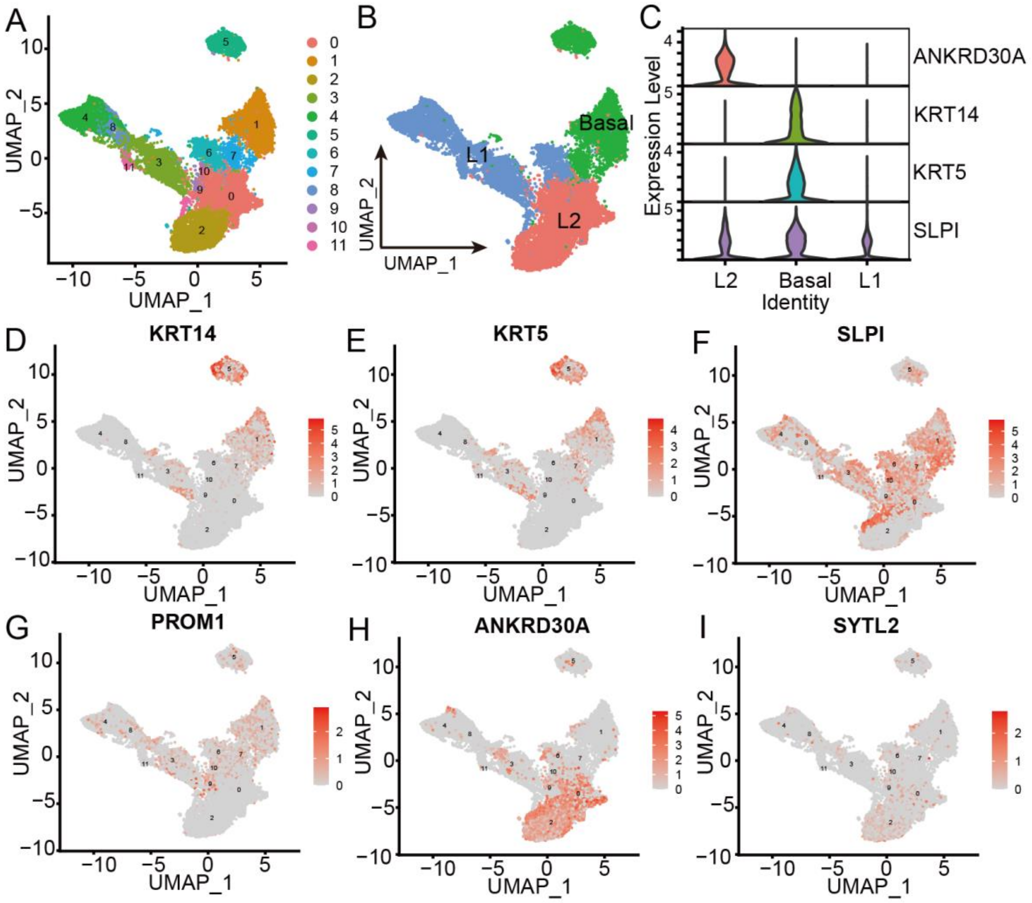

Figure S3. Distribution of epithelial cell subsets in breast cancer.

A: UMAP showed that the epithelial cells of breast cancer were clustered into 11 clusters. B: A: UMAP showed that breast cancer epithelial cells were identified as basal type, luminal 1 and luminal 2 epithelial cells. C: Marker expression corresponding to different epithelial cell subtypes of breast cancer. D~E: UMAP displays marker markers of basal epithelial cells. F~G: UMAP displays marker markers of luminal 1 epithelial cells. H~I: UMAP displays marker markers of luminal 2 epithelial cells.

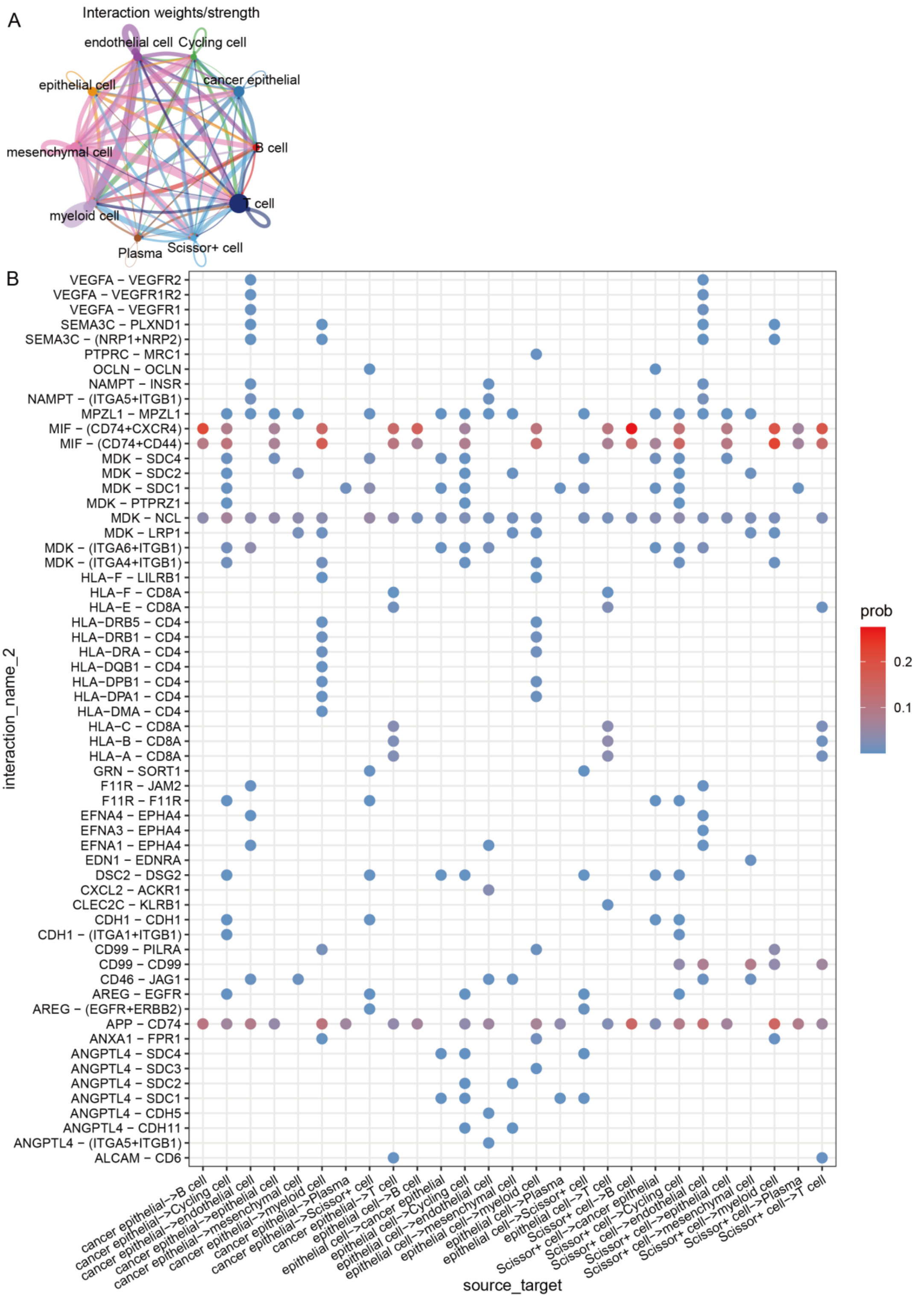

Figure S4. Analysis of intercellular communication in breast cancer microenvironment.

A: The overall distribution of cell communication frequency in different cell types of breast cancer. The thickness of the line represents the frequency of interaction between cells. B: Cell communication between breast cancer epithelial cells (normal epithelial cells, cancer cells, and Scissor+ cancer cells) and other cell types and the distribution of corresponding ligand and receptor pairs.

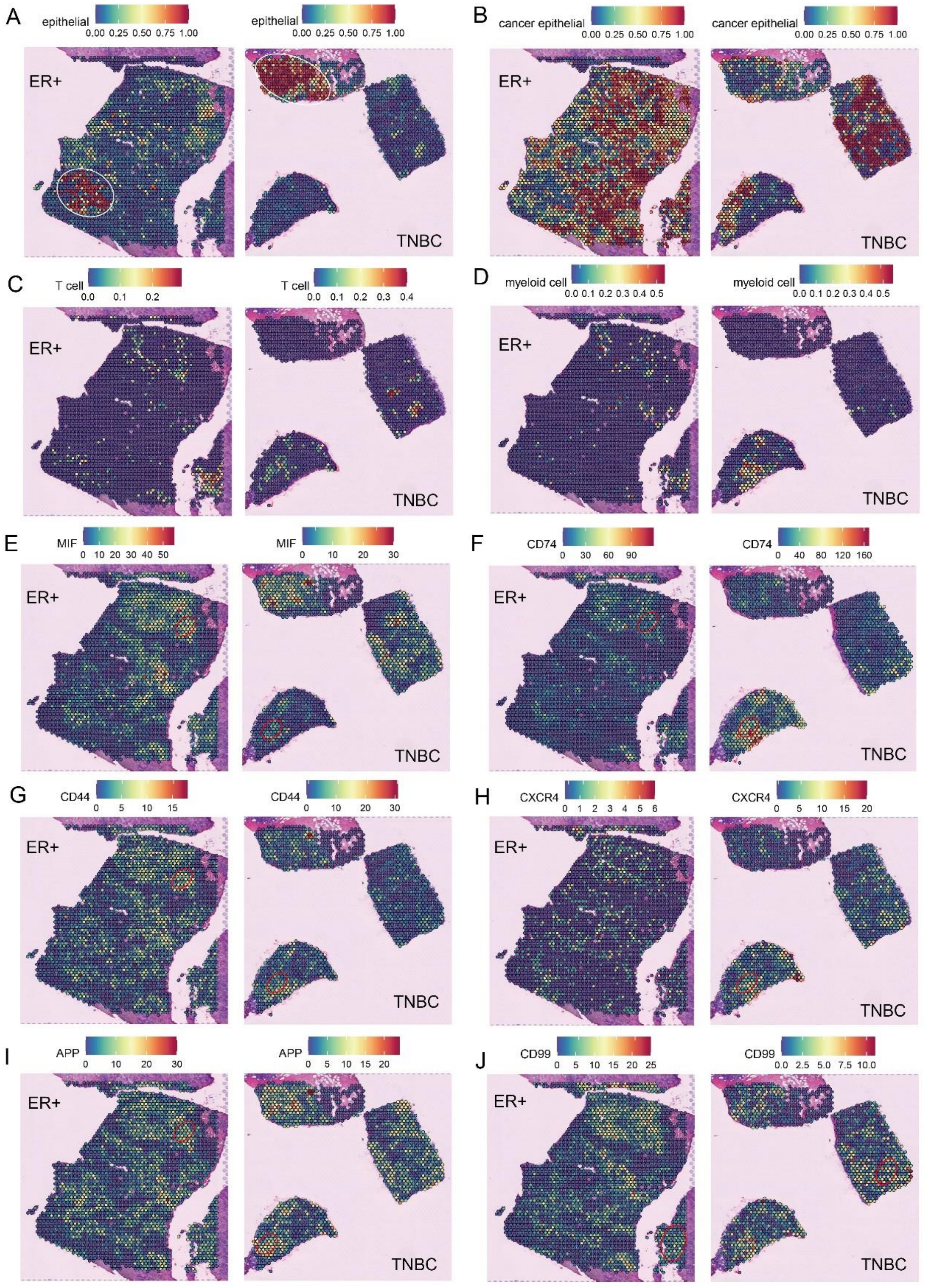

Figure S5. Spatial transcriptome data were used to analyze the spatial distribution of breast cancer cells and their ligands and receptors.

A~D: The spatial distribution of normal epithelial cells, cancer epithelial cells, T cells and myeloid cells in ER+ and TNBC breast cancer tissues. E~J: The spatial expression levels of inflammatory ligand receptors such as MIF, CD74, CD44, CXCR4, and APP in ER and TNBC breast cancer tissue sections. Each spot in the figure records specific spatial location information, and each spot may contain from one to a dozen cells. The color of each spot represents the amount of gene expression in it or the predictive percentage score for each cell type. The locations marked by circles are areas of spatial co-localization. When two or more cell types are spatially adjacent and their ligands and receptors are spatially co located, they are likely to have cellular interactions. In the figure, we can probably see that the simultaneously highly expressed regions of inflammatory ligands and receptors such as APP-CD74, MIF-(CD74+CD44) and CD99-CD99 are mainly distributed in the cancer epithelial cell region or at the border with T cells/myeloid cells (red circle example area). On the contrary, it is rarely observed that these ligands and receptors are highly expressed at the same time in normal epithelial cells (white circle example area).
